# Effect of the Pore Shape and Size of 3D-Printed Open-Porous ABS Materials on Sound Absorption Performance

**DOI:** 10.3390/ma13204474

**Published:** 2020-10-09

**Authors:** Katarina Monkova, Martin Vasina, Peter Pavol Monka, Drazan Kozak, Jan Vanca

**Affiliations:** 1 Faculty of Manufacturing Technologies, Technical University in Kosice, Sturova 31, 080 01 Presov, Slovakia; peter.pavol.monka@tuke.sk (P.P.M.); jan.vanca@tuke.sk (J.V.); 2 Faculty of Technology, Tomas Bata University in Zlin, Nam. T.G. Masaryka 275, 760 01 Zlin, Czech Republic; 3 Faculty of Mechanical Engineering, VŠB-Technical University of Ostrava, 17. listopadu 15/2172, 708 33 Ostrava-Poruba, Czech Republic; 4 Mechanical Engineering Faculty, University of Slavonski Brod, Trg Ivane Brlic-Mazuranic 2, HR-35000 Slavonski Brod, Croatia; dkozak@sfsb.hr

**Keywords:** acrylonitrile butadiene styrene, 3D printing technique, acoustic impedance tube, sound absorption coefficient, volume ratio, excitation frequency of acoustic wave, air space size, material thickness

## Abstract

Noise has a negative impact on our environment and human health. For this reason, it is necessary to eliminate excessive noise levels. This paper is focused on the study of the sound absorption properties of materials with open-porous structures, which were made of acrylonitrile butadiene styrene (ABS) material using additive technology. Four types of structures (Cartesian, Octagonal, Rhomboid, and Starlit) were evaluated in this work, and every structure was prepared in three different volume ratios of the porosity and three different thicknesses. The sound absorption properties of the investigated ABS specimens were examined utilizing the normal incidence sound absorption and noise reduction coefficients, which were experimentally determined by the transfer function method using a two-microphone acoustic impedance tube. This work deals with various factors that influence the sound absorption performance of four different types of investigated ABS material’s structures. It was found, in this study, that the sound absorption performance of the investigated ABS specimens is strongly affected by different factors, specifically by the structure geometry, material volume ratio, excitation frequency of an acoustic wave, material’s thickness, and air space size behind the tested sound-absorbing materials.

## 1. Introduction

Sound absorption can be defined as a decrement of sound energy upon contact of sound waves with sound absorbing material, such as, e.g., walls, ceilings, floors, or other subjects, which causes the sound to not be reflected back into space [1,2]. Sound absorption can be a particularly important factor not only for spaces such as concert venues, cinemas, and theatres but also, e.g., for schools, lecture aulas, and for many other materials of daily applications. This also became important for various fields of industrial practice, e.g., for the correction of noise that may arise from machines, railroad cars, or computer server array cooling fans in search engines or cloud computing, or for the components belonging to the automotive industry [3,4,5,6].

Sound absorption efficiency is mostly connected with the thickness of the bulk material [7,8]. On the other hand, to improve the effectivity of sound absorbent materials at lower frequencies, this can be compensated by the air space behind the acoustic board [1]. However, the homogeneous fully infilled materials are not sufficient for good sound absorption, but better sound absorption characteristics are usually shown for porous materials such as foams and fibers [9,10,11,12]. When sound waves propagate inside a porous material, they pass through the fibers (thin walls) connecting the pores and, as a result of viscous losses, the acoustic energy is converted into heat. To dissipate acoustic energy, air movement inside the porous material is required, which causes the material located near the fixed boundary (where the particle velocity is zero) to become ineffective [13,14]. The optimum sound absorption coefficient related to an optimum viscous boundary layer thickness of a micro lattice metamaterial has been analyzed by Cai et al. [15]. 

Deshmukh et al. [16] compared the sound properties of the bulk samples with the samples of lattice sandwich structures. It was found that the lattice structures exhibit excellent insulating sound properties compared to the bulk samples. The different geometries of porous structures produced by 3D printing technique have been studied from the aspect of acoustic absorption. Excellent mechanical properties are ensured by the face-centered cubic (FCC) core within the additively produced structures. Wang et al. [17] and Jiménez et al. [18] investigated highly porous materials supported by rigid supports of multiple layers with a solid frame and theoretically analyzed various configurations of porous materials and their increasing complexity, namely one layer of highly porous material, one layer of highly porous air space material, and a multi-layer structure. A porous pyramid sandwich structure was studied by Liu et al. [19] that was characterized by a good ratio of strength and stiffness to weight. Due to its good mechanical properties, such a porous material is used in applications ranging from lightweight structures to aircraft components [20].

Double-porosity materials were studied by Olny and Boutin [21] to control the absorption coefficient in the desired frequency range, in which periodic porous material at the micro level was examined using a homogenization technique and then applied to heterogeneous triple-range materials. The behavior of the specific, periodically arranged cavities with a cylindrical geometry in porous materials was investigated at the microscopic level by Roh and Yoon [22], at which the losses of thermal and viscous characters were taken into account. Lagarrigue et al. [23] came with the idea of incorporating the rigid inclusions in a porous material to be tunable and for it to be possible to improve their sound absorption properties at the low-frequency scope. Cellular materials with several types of inclusions differing in shape was studied by Groby et al. [24], while Xu et al. [25] dealt with an optimum design for the propagation of acoustics in the exact material layer inside the cavity using the bounded element method.

Numerical analysis of porous layer properties and their optimization by linking effective acoustic properties and geometry were discussed in several available studies. While Bulvert et al. [26] used a non-linear conjugate gradient algorithm for optimization, Krödel et al. [27] used Biot’s theory that confirmed a good match of a predicted sound wave speed with the experimental measures within an analysis of the acoustic response of periodic millimeter-sized microlattices immersed in water. An analytical model of obtaining the coefficient of loss of admitted sound in a cylindrical box made of porous materials was presented by Gohari et al. [28]. The measured values have shown that when the length of the porous structure is increased, the loss of sound transmission approximates that of the infinite shells. They also have found out that by changing the length of the poroelastic roller, the amount of transmitted sound was reduced. However, better sound insulation properties are obtained in the low-frequency band when the shell radius is reduced. Numerical analysis and experimental research of the propagation and loss of sound in porous materials in pipelines using regular fixed diffusers to increase transmission loss by Bragg diffraction was carried out by Jena and Qiu [29]. The most preferred method for calculating sound propagation through homogeneous sandwich materials as well as porous materials with isotropic properties in a pipe that is below the cut-off frequency is the transfer matrix method [30,31], while for simulating the behavior of complex structures and inclusions with complex shape in a wide frequency range, it is appropriate to use numerical models [32,33]. 

There are many sound-absorbent materials, including various plastics and porous fibers, the development of which has advanced rapidly in recent years. They are used to effectively control noise or to improve the acoustic conditions by shortening the echo time in the room [34,35,36]. The important aspects of the applicability of sound-absorbent materials are not only their effective noise absorption and insulation but also their structural stability, environment- and health-friendly properties, as well as their suitability for mass production [37,38]. These requirements caused a significant part of the research to be focused on investigating the properties of polymer composites and polycarbonate. 

As the complex structures for a sound absorption application are easily manufactured by a 3D printing technique, it is very interesting to deal with these specific properties of the materials usually used within this technology. One of the most widely used plastics available in filaments for additive manufacturing is acrylonitrile butadiene styrene (ABS). However, only a few studies [39,40,41] have been focused on the investigation of the sound absorption properties of components produced from ABS material.

The main benefit of ABS as a material is its possibility to combine the toughness of the polybutadiene rubber with the rigidity and strength of acrylonitrile and styrene polymers. This material is resistant to chemical degradation either by alkaline or acidic agents. For most applications, ABS can be used between −20 to 80 °C [42]. In addition to the good mechanical properties, ABS material also shows good electrical insulating characteristics over a wide range of frequencies, even in high humidity and high temperature environments, making this material appropriate as a protecting material for electrical components such as chips and cables [43]. The next advantageous property of ABS is that it belongs to the category of thermoplastics that means that even with repeated heating and cooling, it does not degrade [44]. This property classifies material as suitable for recycling. It is an amorphous solid, which technically means that ABS hasn’t a melting point [45]. It is flammable at open flames or in case of its exposure to very high temperatures [46]. It is widely applied at daily used things and household appliances such as, e.g., knife and plier handles, for the manufacture of pipes for water treatment and distribution, for air conditioning, in the food industry, for the manufacture of furniture, etc. It is useful for the production of complex and precise shaped components such as musical instruments, automotive parts, medical devices, and others. As it is widely used in many applications, it is also worthy to discover the sound absorption properties of the structures made from this ABS plastic material to use it for such a purpose.

Generally, unperforated hard solid materials (e.g., concrete, stone, glass, and plastics) are not suitable for noise reduction. Rather, they can be used as sound reflective materials. The open-porous materials manufactured using 3D printing technology are applicable for products in the case if it is possible to ensure their both mechanical stiffness and sound absorption in practice. The goal of this work is to study various factors affecting the sound absorption performance of 3D-printed open-porous ABS materials which were produced with different geometry of pores inside of lattice metamaterials with different thicknesses and with different volume ratios. Based on the abovementioned and also the authors’ best knowledge, it can be stated that a study of such porous structures produced by the additive technology from the acrylonitrile butadiene styrene (ABS) material and the effects of several parameters (the pore shape within a structure, volume ratio *V_r_* of the material within the sample, sample thickness *t*, and air space size *a*) on their sound absorption properties have not yet been investigated and presented from such a complex point of view.

## 2. Materials and Methods 

### 2.1. Characteristics of ABS Samples and Their Production 

The samples for the research have been produced from acrylonitrile butadiene styrene (ABS) material (Smart Materials 3D, Alcala la Real, Spain).

In this study, the plastic material ABSplus-P430 Ivory was used as a filament with a diameter of 1.75 mm, while the Fused Filament Fabrication (FFF) technique was employed as the additive manufacturing technology.

The main advantage of additive technology is the feasibility to manufacture complex-shaped components with cavities that is not achievable by another type of technology. Such specific materials are also used for sound absorption applications, at which the size and shape of the pores affect the properties of the material. The volume of material separating individual pores in the structure of the material is given by the volume ratio *V_r_* that is expressed by the Equation (1) [47]:

(1) $$V_{r}\left(\%\right)=\frac{V_{S}}{V_{T}}\times 100$$

where *V_S_* is the volume of material used to make the structure, and *V_T_* is the volume of the whole solid body.

Virtual models were generated in PTC Creo software (Parametric Technology Corporation Inc., Boston, MA, USA), version 6. This CAD/CAM system differs from version 5 by offering automatic generation of some types of cellular structures, which is very advantageous for creating a product for application in real practice when the component has a complex shape and it is necessary to fill its core with a light- or sound-absorbing structure. By defining the basic conditions, such as the location of the base cell, its size, location of the struts, etc., the software automatically fills the specified space with the defined structure. Hence, the design of types of structures and their geometries in the research was related to the software offer, while at the same time, the authors tried to focus on atypical and little-studied structures such as Rhomboid, Starlit, or Octagonal and compare their behavior with a simple but relatively frequently used Cartesian structure.

Due to the requirements of the testing equipment (see Section 2.2.3), the samples were designed in a cylindrical shape with an external diameter of 29 mm. A continuous layer of the ABS material of 2 mm thick formed a shell enclosing the inner lattice structure. The lattice structure in the core of every specimen was created in such a way that so-called a “basic cell” has been patterned in the radial direction and the *z*-axis direction, or in all three orthogonal directions along the *x*, *y*, or *z* axes, while the *z*-direction was always parallel to the axis of the cylindrical shape of the specimen. Each cell type contains not only all the outer beams (horizontal and vertical) but also the inner angular struts.

**Figure 1:**
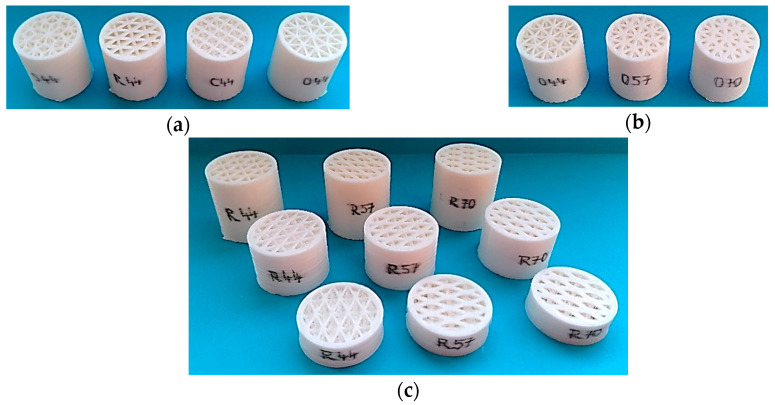
The examples of investigated samples: (**a**) different shapes of porous structures; (**b**) different volume ratios *Vr* of the Octagonal structure; (**c**) a set of the specimens of the Rhomboid structure with different sample thicknesses *t* = 1, 2, and 3 cm, and with different sample volume ratios *V_r_* = 44, 57, and 70%.

In this study, 36 types of lattice structures varied in shape, volume ratio *V_r_*, and thickness *t* were experimentally tested. They were named according to their shape of an internal structure as Starlit, Rhomboid, Cartesian, and Octagonal as they are shown in Figure 1a.

Every shape of the specimen was made in three different volume ratios of 44, 57, and 70% (see Figure 1b), and in three different thicknesses with the values of 1, 2, and 3 cm (Figure 1c).

The choice of volume ratios in the presented research was therefore mainly connected with the possibility to define the same volume of material in the software for all 4 types of structures. The same volume ratio was determined as a basic parameter for comparing the sound absorption properties of individual structures and for evaluating the efficiency of the use of plastic material in production, and it has been controlled over the diameter of the pore-enveloping strut. The specification of the smallest volume fraction *V_r_* = 40% was also related to the technological conditions because the production of structures with a lower volume fraction was either not possible or the samples were of poor quality. When selecting the largest considered volume fraction *V_r_* =70%, the aspect of the practical character was also taken into account, in which the saving of material in combination with the given production technology would show not only an economic but also time and ecological effects.

The samples were 3D-printed at the orientation of the cylindrical axis *z* normal to the building platform *xy*. The next properties of the individual internal structures are presented in Table 1.

Due to the test of sound absorption properties being non-destructive, it was enough to make only one piece of every type of the specimens; that means 36 pieces of the samples with 4 types of porous structures at 3 volume ratios *V_r_* and 3 different thicknesses *t*.

The 3D printer Prusa i3 Mk2 (Prusa Research a.s., Prague, Czech Republic) was used for specimen production. During printing, the material prepared in the form of a plastic fiber is unwound from a spool and is guided inside the extruder. The nozzle melts the filament (temperature of the nozzle has been 255 °C) and extrudes it onto a build platform with a temperature of 100 °C. Both the nozzle and the platform are controlled by a computer which converts the dimensions of the body into *x*, *y*, and *z* coordinates so that the print head moves correctly in relation to the building platform. As is usual with the FFF method, the printhead also moves over the base to “draw” the cross-sectional shape of the printed body on the base plate. The rate of the nozzle in this research was 40 mm/s at the shape circumference and 30 mm/s inside the cross-section of a sample. This thin layer of plastic cools and hardens very quickly bonded to the layer beneath it. When the layer is finished, the base is moved to make space for another layer of material. When an object is taken off the FFF printer, its support materials are usually removed if they have been used. In the case of investigated samples, no supports were used.

In contrast to the authors’ initial already published research [48], in which only 3 types of structures with one volume ratio *V_r_* = 57% were used to assess and compare sound properties, within the presented research a different nozzle size and a different layer height of the applied material were used for the samples production. These technological conditions affect not only the quality of the finished product but also its mechanical properties [49,50]. While in the previous research, a nozzle with a diameter of 0.6 mm and a height of the applied layer of 0.3 mm was employed, in this case, the samples were made with a nozzle with a diameter of 0.4 mm and a layer height of 0.254 mm. Although the production time of the samples was extended by almost 60%, the quality of the samples (in terms of shape, purity, and compactness of the printout) has already increased at first glance.

### 2.2. Measurement Methodology

#### 2.2.1. Sound Absorption Coefficient

A material’s ability to damp sound is expressed by the sound absorption coefficient that measures the fraction of incident sound energy which is absorbed on the surface of the sound-absorbing material. The sound absorption coefficient *α* (−) is defined by the equation [51], Equation (2):

(2) $$\alpha =\frac{P_{A}}{P_{I}}$$

where *P_A_* is the absorbed (dissipated) acoustic power and *P*_I_ is the incident acoustic power. Generally, the material’s ability to absorb sound is affected by different aspects, such as the sound-absorbing material type, excitation frequency of an acoustic wave, thickness, pore geometry, air flow resistivity, density, temperature, and humidity. The airflow resistivity is defined by the ratio of the static pressure difference between two sides of the tested material to the line speed of the airflow which stably passes through the porous material. It is well known that an increase of the airflow resistivity improves the absorption performance in the whole frequency range but only until an intermediate value [52]. An overly acoustically resistant porous material exhibits low sound absorption behavior and prevents the propagation of acoustic waves into the interior of the material. Nevertheless, the smaller flow resistance of porous materials will less efficiently convert sound energy into heat which, again, leads to the low sound absorption performance. For this reason, it is necessary to ensure an optimum flow resistance of the porous material [52,53].

#### 2.2.2. Noise Reduction Coefficient

As mentioned above, the sound absorption properties of sound-absorbing materials are strongly influenced by the excitation frequency. This phenomenon is given by the noise reduction coefficient (NRC), which is specified as the arithmetical average of measured values of the sound absorption coefficient in the central frequency bands 250, 500, 1000, and 2000 Hz [54,55], Equation (3):

(3) $$\mathrm{NRC}=\frac{1}{4}\cdot \left(\alpha_{250}+\alpha_{500}+\alpha_{1000}+\alpha_{2000}\right).$$

#### 2.2.3. Sound Absorption Performance

**Figure 2:**
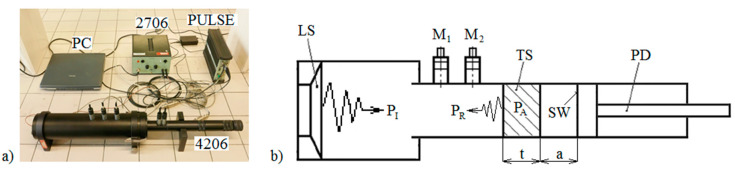
View of the experimental setup for measuring frequency dependencies of the sound absorption coefficient (**a**) and a scheme of the two-microphone acoustic impedance tube (**b**). Legend of the abbreviations: *a*—air space size; LS—loudspeaker; M_1_, M_2_—measuring microphones; *P_A_*—absorbed acoustic power; *P_I_*—incident acoustic power; *P_R_*—reflected acoustic power; PD—piston disk; TS—Tested Sample; SW—solid wall; *t*—sample thickness.

Experimental measurements of the normal incidence sound absorption coefficient of the studied 3D-printed open-porous ABS materials in the frequency range of 200–6400 Hz were performed employing a two-microphone acoustic impedance tube (BK 4206), a dynamic signal PULSE multi-analyzer (BK 3560-B-030), and a power amplifier (BK 2706). The view of the experimental setup (Brüel & Kjær, Nærum, Denmark) for measuring frequency dependencies of the sound absorption coefficient is shown in Figure 2a.

Frequency dependencies of the normal incidence sound absorption coefficient of the investigated ABS specimens of a given thickness *t* (ranging from 1 to 3 cm) were experimentally measured for various air space sizes *a* (ranging from 0 to 12 cm) behind the tested samples. The air space size was set by means of the movable piston disk, as shown in Figure 2b.

Experimental measurements of the normal incidence sound absorption coefficient were performed by means of the two-microphone transfer function method [56] based on the standing wave principle. This method has been standardized by the International Organization for Standardization as ISO 10534-2. Based on this method, the normal incidence sound absorption coefficient *α* is given by Equation (4) [57]:

(4) $$\alpha =1-{\left|r\right|}^{2}$$

where *r* is the sound reflection factor at normal incidence that is defined as Equation (5):

(5) $$r=e^{2ikx_{1}}\cdot \frac{H-H_{I}}{H_{R}-H}$$

where *k* is the complex wave number, *x*_1_ is the distance between the microphone M_1_ from the reference sample plane (*x* = 0), *H* is the complex acoustic transfer function, *H_I_* is the transfer function of the incident wave, and *H_R_* is the transfer function of the reflection wave. The transfer functions are defined by the following equations, Equations (6)–(8):

(6) $$H=\frac{p_{2}}{p_{1}}=\frac{e^{ikx_{2}}+r\cdot e^{-ikx_{2}}}{e^{ikx_{1}}+r\cdot e^{-ikx_{1}}}$$

(7) $$H_{I}=e^{-k\cdot \left(x_{1}-x_{2}\right)i}$$

(8) $$H_{R}=e^{k\cdot \left(x_{1}-x_{2}\right)i}$$

where *x*_2_ and is the distance between the microphone M_2_ from the reference sample plane (*x* = 0), and *p*_1_ and *p*_2_ are the complex acoustic pressures that were measured by means of the microphones M_1_ and M_2_. The complex wave number *k* is defined as Equation (9):

(9) $$k=k^{\prime }-ik^{\prime \prime }$$

where *k*^′^ and *k*^″^ are the real and imaginary parts of the complex wave number that are expressed by the equations, Equations (10) and (11):

(10) $$k^{\prime }=\frac{2\pi \cdot f}{c}$$

(11) $$k^{\prime \prime }=\frac{4f}{c}\cdot ln\left(\frac{{∆}_{n}}{2}+\sqrt{\frac{{∆}_{n}^{2}}{4}+1}\right)$$

where *f* is the excitation frequency, *c* is the speed of sound, and Δ*_n_* is the dimensionless quantity that is obtained from the experimentally measured acoustic pressure amplitudes in two neighboring local minima *x_min_* (numbered *n* and *n* + 1) and the local maximum *x_max_* (numbered *n*) between the local minima, Equation (12):

(12) $${∆}_{n}=\frac{\left|p\left(x_{min,\;n+1}\right)\right|-\left|p\left(x_{min,\;n}\right)\right|}{\left|p\left(x_{max,\;n}\right)\right|}$$

where *n* is the integer (i.e., *n* = 0, 1, 2, …).

## 3. Results

This section deals with various aspects that affect the sound absorption performance of the studied lattice structures made by additive technology from ABS material, which is marked as follows: Firstly, the sample specification is given by the geometry of the structure (see Table 1). Thereafter, the label of the sample is made up of three numbers. The first one defines the volume ratio *V_r_* (in %). The second number represents the sample thickness *t* (in cm). Finally, the third number denotes the air space size *a* (in cm) behind the Tested Sample TS inside the acoustic impedance tube (see Figure 2b).

### 3.1. Frequency Dependencies of the Sound Absorption Coefficient

#### 3.1.1. Influence of Pore Shape

**Figure 3:**
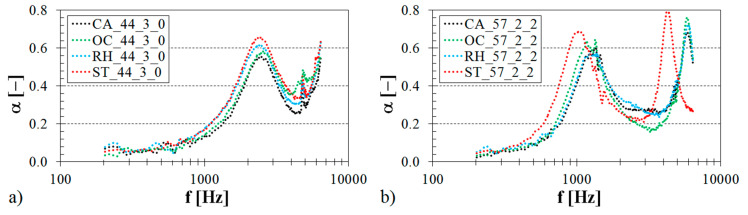
Influence of the pore geometry of the 3D-printed ABS material on the frequency dependencies of the sound absorption coefficient: (**a**) volume ratio *V_r_* = 44%, sample thickness *t* = 3 cm, air space size *a* = 0 cm, (**b**) volume ratio *V_r_* = 57%, sample thickness *t* = 2 cm, air space size *a* = 2 cm.

The structure type of 3D-printed ABS materials is affected by pores of various shapes and sizes and has a substantial effect on their sound absorption performance. The influence of the pore geometry on the sound absorption behavior of the open-pore ABS materials is illustrated in Figure 3.

The effect of the structure type of the tested ABS samples (with *V_r_* = 44% and *t* = 3 cm) fixed directly on the solid wall SW (i.e., with *a* = 0 cm) inside the acoustic impedance tube (see Figure 2b) is shown in Figure 3a. Similarly, the effect of the structure type on frequency dependencies of the sound absorption coefficient for the ABS samples of the same volume ratio (i.e., *V_r_* = 57%) and the same thickness (i.e., *t* = 2 cm) placed at a distance of 2 cm from the solid wall is demonstrated in Figure 3b. It is evident that the effect of the structure type on the sound absorption performance was generally negligible for low-frequency acoustic waves, specifically at *f* < 700 Hz (see Figure 3a) and at *f* < 400 Hz (see Figure 3b). Furthermore, it can be seen in a wide frequency range that better sound absorption performance was noticed for the ABS samples, which were made with the Starlit structure. Conversely, the ABS samples manufactured with the Cartesian structure (see Figure 3a) are characterized by a lower ability to absorb sound compared to the other ABS structures examined. The effect of the structure type of the studied ABS materials is connected with the airflow resistivity that is the most important parameter affecting the sound absorption performance of porous materials [58]. As shown in Table 1, the Starlit structure type is specified by more complex pore geometry in comparison with the other open-porous structure types. This phenomenon is accompanied by multiple sound reflections and by higher internal friction during the acoustic wave propagation through this porous structure type. For these reasons, the Starlit structure exhibited a more efficient conversion from sound energy into heat than the other investigated open-porous structures. This effect was observed in the frequency ranges of 1.7–4.1 kHz (see Figure 3a), 0.4–1.2 kHz (see Figure 3b), and 3.3–4.0 kHz (see Figure 3b). Contrariwise, the 3D-printed ABS specimens prepared with the Cartesian structure were characterized by a lower ability to dampen sound in the frequency ranges of 1.0–6.4 kHz (see Figure 3a) and 0.8–1.2 kHz (see Figure 3b) compared to the other open-porous structure types.

#### 3.1.2. Influence of Volume Ratio

**Figure 4:**
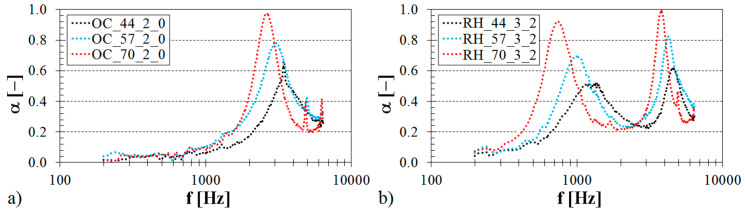
Influence of the sample volume ratio on the frequency dependencies of the sound absorption coefficient for the tested ABS samples: (**a**) Octagonal structure, sample thickness *t* = 2 cm, air space size *a* = 0 cm; (**b**) Rhomboid structure, sample thickness *t* = 3 cm, air space size *a* = 2 cm.

The volume ratio (or the relative density), which increases with a decrease in the pore size, generally has a big influence on the airflow resistivity [59,60] and, thus, on the sound damping properties of the examined open-porous structures. The relative density, which increases with an increase in the strut diameter (see Table 1), increases the mass and, thus, the mechanical stiffness of the tested ABS materials [61]. Generally, higher density open-porous materials have better sound absorbing properties than lower density materials with open pores [58,62,63]. The influence of the sample volume ratio on the frequency dependencies of the sound absorption coefficient for two various types of 3D-printed structures (i.e., Octagonal and Rhomboid) is demonstrated in Figure 4.

It is visible that the sound absorption performance of the tested ABS samples generally increased with an increase in the volume ratio at low excitation frequencies. In the case of the ABS sample measuring 2 cm in the thickness (see Figure 4a), which was produced with the Octagonal structure and was fixed directly on the solid wall SW inside the impedance tube (see Figure 2b), higher values of the sound absorption coefficient were observed for the volume ratio *V_r_* = 70% in the frequency range of 0.7–2.0 kHz. If the ABS sample of the same volume ratio measuring 3 cm in the thickness was made with the Rhomboid structure and positioned at a distance of 2 cm from the solid wall (see Figure 4b), a positive effect of the volume ratio on the sound absorption performance was observed in the frequency ranges of 200–880 Hz and 1.5–3.0 kHz. Conversely, the abovementioned ABS samples, which were produced with the smallest volume ratio (i.e., with *V_r_* = 44%), exhibited higher values of the sound absorption coefficient in the frequency ranges of 3.9–4.8 kHz (see Figure 4a) and 1.3–2.5 kHz (see Figure 4b) compared to the same ABS samples that were produced with higher volume ratios (i.e., with *V_r_* = 57% and *V_r_* = 70%). It can be noticed in the case of the OC_70_2_0 sample type (see Figure 4a) that frequency bands corresponding to a given number of peaks of the sound absorption coefficient vary in their width. Generally, wider frequency bands are obtained at higher values of the sound absorption coefficient. Therefore, the first frequency band (*α_max_ ≈* 0.97) is characterized by the broad peak compared to the second and third frequency bands (*α_max_ ≈* 0.41) that appear narrower.

#### 3.1.3. Influence of Material Thickness

**Figure 5:**
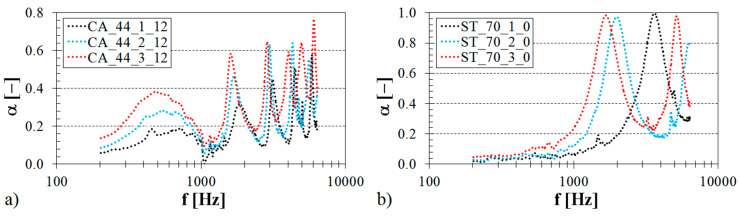
Influence of the sample thickness on the frequency dependencies of the sound absorption coefficient for the tested ABS samples: (**a**) Cartesian structure, volume ratio *V_r_* = 44%, air space size *a* = 12 cm; (**b**) Starlit structure, volume ratio *V_r_* = 70%, air space size *a* = 0 cm.

The material thickness also belongs to the important factors affecting the sound absorption performance of the investigated 3D-printed open-porous ABS samples. Figure 5 illustrates the influence of different sample thickness when the studied sample is positioned at a distance of 12 cm from the solid wall (see Figure 5a) or is mounted directly on the solid wall (see Figure 5b). 

It was found that the sound absorption performance significantly increased with an increase in the material thickness toward lower excitation frequencies. It should also be noted that frequency dependencies of thicker porous materials are generally characterized by wider frequency bands compared to thin porous materials. Therefore, the application of very thin porous materials with air-gap construction to absorb sound is suitable only for narrow frequency bands depending on the sample distance from the solid wall (more in Section 3.1.4). Nevertheless, the material thickness of 3D-printed products may affect their printing times and production costs [64,65]. Because the printing process conditions of a given open-porous structure and the ground plane dimensions (i.e., samples with an outer diameter of 29 mm) of the tested 3D-printed samples were identical, the increasing material thickness results in an increase in the printing time and the manufacturing costs of the 3D printing technology [66]. For this reason, this method is not effective to damp noise.

#### 3.1.4. Influence of Air Space Size

**Figure 6:**
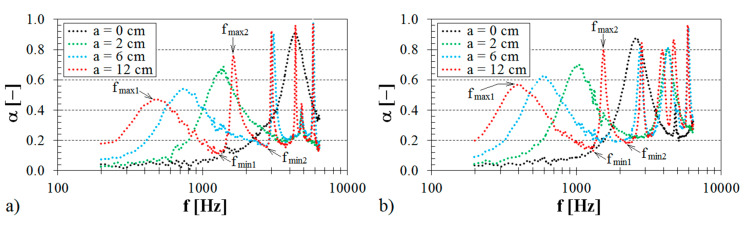
Influence of the air space size on the frequency dependencies of the sound absorption coefficient for the tested ABS samples: (**a**) Rhomboid structure, volume ratio *V_r_* = 44%, sample thickness *t* = 1 cm; (**b**) Starlit structure, volume ratio *V_r_* = 57%, sample thickness *t* = 2 cm.

The air space size between the Tested Sample TS and the solid wall SW inside the impedance tube (see Figure 2b) is another factor which strongly affects the sound absorption performance of porous materials. Figure 6 shows the effect of the air space size on sound absorption behavior for two different ABS samples.

It is evident that the frequency dependencies of the sound absorption coefficient have oscillating waveforms with a certain number of minima and maxima of the sound absorption coefficient at the corresponding excitation frequencies *f_min_* and *f_max_*. This phenomenon is related to the reflections of acoustic waves from the solid wall inside the acoustic impedance tube and to the wavelength of sound *λ*, which is defined by the ratio of the speed of sound to the frequency [67]. The maximal acoustic pressure is reached at the solid wall surface. Conversely, the air particle velocity is equal to zero at the wall. Generally, the sound absorption maximum is reached for the maximum air particle velocity and zero acoustic pressure. For these reasons, the maximum sound absorption coefficient is obtained, when the air space size is equal to the odd multiples of ¼ wavelength [68,69] at the excitation frequencies, Equation (13):

(13) $$f_{max}=\frac{c\cdot \left(2n+1\right)}{4l}$$

where *n* is an integer (*n* = 0, 1, 2, …) and *l* is the sample distance from the solid wall, which is given by the formula shown in Equation (14):

(14) $$l=a+\frac{t}{2}$$

Similarly, the minimum sound absorption coefficient is obtained, when the air space size is equal to the even multiples of ¼ wavelength at the excitation frequencies, Equation (15):

(15) $$f_{min}=\frac{c\cdot n}{2l}$$

**Figure 7:**
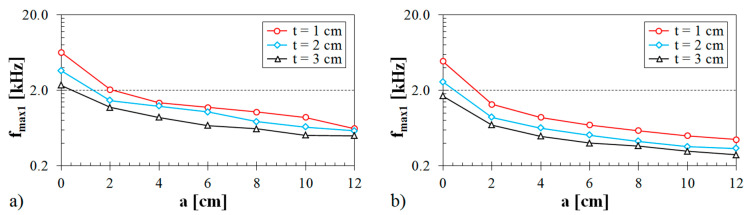
Dependencies of the primary sound absorption maxima on the air space size at various thicknesses of the tested ABS samples: (**a**) Rhomboid structure, volume ratio *V_r_* = 44%; (**b**) Octagonal structure, volume ratio *V_r_* = 70%.

It is evident from the Equations (13) and (15) that the excitation frequencies, at which the sound absorption coefficient reaches the maximum and minimum values, decrease with an increase in the distance *l* and the air space size *a*, respectively. This phenomenon was also confirmed by the measured frequency dependencies of the sound absorption coefficient, as shown in Figure 6. Therefore, a higher air space size between the porous sample and the solid wall inside the impedance tube generally leads to improving the sound absorption behavior of porous materials in the low frequency range [68,70]. The effect of the air space size *a* on the primary excitation frequency *f_max_*_1_, which corresponds to the primary sound absorption maxima *α_max_*_1_, is depicted in Figure 7.

It can be seen that the primary excitation frequency decreased with an increase in the air space size independently of the specimen thickness. Therefore, instead of increasing the sound absorber thickness, which means adding more 3D-printed materials, increasing the air space size is more effective in terms of sound absorption.

#### 3.1.5. Influence of Excitation Frequency

The excitation frequency of acoustic wave propagation in solids is one of the significant factors affecting the sound absorption performance of the tested 3D-printed open-porous ABS samples. As shown in Figure 3, Figure 4, Figure 5 and Figure 6, the highest values of the sound absorption coefficient were observed at certain excitation frequencies depending on the structure type, volume ratio, thickness, and air space size of the investigated 3D-printed open-porous ABS samples. The maximum values of the sound absorption coefficient *α_max_* for each type of the ABS structure type and the corresponding excitation frequency *f_max_*, including volume ratio, sample thickness and air space size, are shown in Table 2.

It is obvious that the absorption maxima were generally obtained for the ABS samples that were produced with the highest volume ratio and higher sample thickness and were located at a greater distance from the solid wall inside the acoustic impedance tube. It is also evident from Figure 3, Figure 4, Figure 5 and Figure 6 that the sound absorption properties of the ABS samples were poor at low frequencies, which is a common property of porous materials [71,72].

### 3.2. Noise Reduction Coefficient

As stated above, the excitation frequency of acoustic wave propagation in solids has a substantial effect on the sound absorption performance. Therefore, the noise reduction coefficient, which is defined by Equation (3), was introduced in order to describe an average sound absorption performance of tested sound absorbing materials. This section deals with different factors affecting the noise reduction coefficient of the investigated 3D-printed open-porous ABS materials.

#### 3.2.1. Influence of Air Space Size

**Figure 8:**
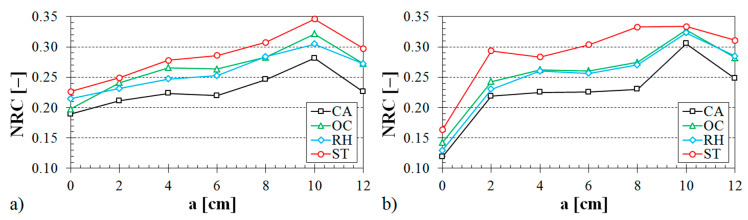
Influence of the 3D-printed ABS material structure on the noise reduction coefficient vs. air space size for the tested ABS samples: (**a**) volume ratio *V_r_* = 44%, sample thickness *t* = 3 cm; (**b**) volume ratio *V_r_* = 57%, sample thickness *t* = 2 cm.

**Figure 9:**
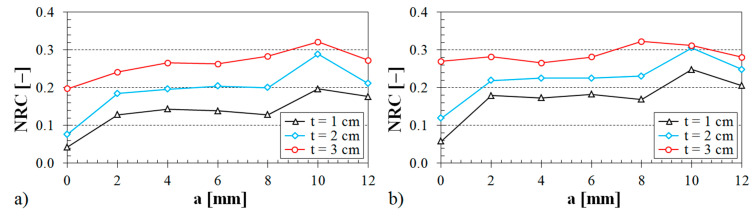
Influence of the material thickness on the noise reduction coefficient vs. air space size for the tested ABS samples: (**a**) Octagonal structure, volume ratio *V_r_* = 44%; (**b**) Cartesian structure, volume ratio *V_r_* = 57%.

**Figure 10:**
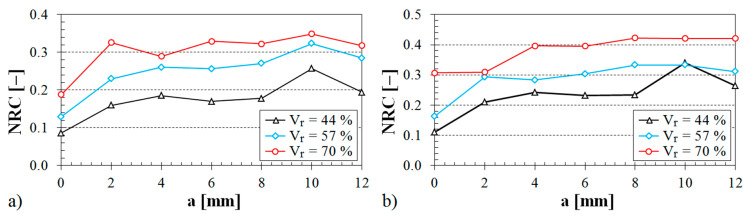
Influence of the volume ratio on the noise reduction coefficient vs. air space size for the tested ABS samples: (**a**) Rhomboid structure, sample thickness *t* = 2 cm; (**b**) Starlit structure, sample thickness *t* = 1 cm.

The influence of the air space size between the tested sample and the solid wall inside the impedance tube on the noise reduction coefficient is shown in Figure 8, Figure 9 and Figure 10.

The influence of the structure type on the sound absorption behavior is shown in Figure 8. It is obvious from this evaluation that the specimens made from the ABS material with the Starlit structure had higher values of the noise reduction coefficient independently of the air space size. Therefore, these samples made with the Starlit structure showed a better capability to dampen noise in comparison with other structure types. Conversely, the lowest values of the noise reduction coefficient were obtained for the samples that were prepared with the Cartesian structure. It was also found from the dependencies of the noise reduction coefficient on the air space size that the sample thickness (see Figure 9) and the volume ratio (see Figure 10) have a positive effect on the sound absorption behavior of the investigated ABS samples. 

#### 3.2.2. Influence of Material Thickness

**Figure 11:**
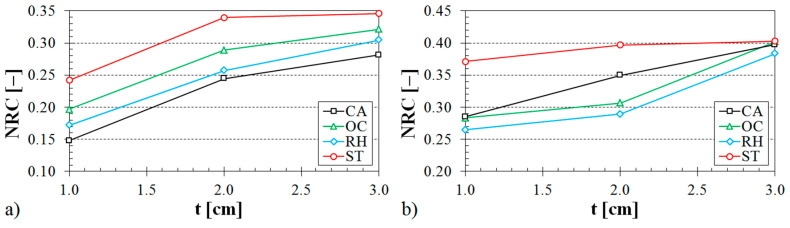
Influence of the 3D-printed ABS material structure on the noise reduction coefficient vs. material thickness for the investigated ABS samples: (**a**) volume ratio *V_r_* = 44%, air space size *a* = 10 cm; (**b**) volume ratio *V_r_* = 70%, air space size *a* = 4 cm.

**Figure 12:**
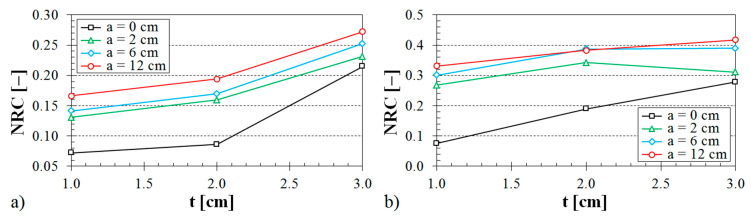
Influence of the air space size on the noise reduction coefficient vs. material thickness for the investigated ABS samples: (**a**) Rhomboid structure, volume ratio *V_r_* = 44%; (**b**) Cartesian structure, volume ratio *V_r_* = 70%.

**Figure 13:**
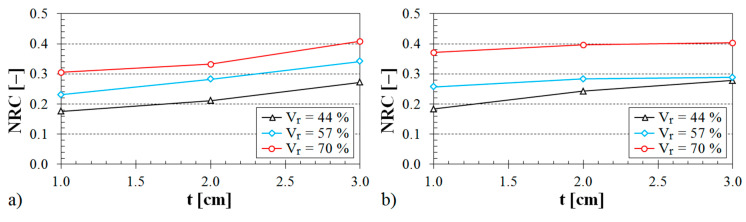
Influence of the volume ratio on the noise reduction coefficient vs. material thickness for the investigated ABS samples: (**a**) Octagonal structure, air space size *a* = 12 cm; (**b**) Starlit structure, air space size *a* = 4 cm.

The influence of the ABS sample thickness on the noise reduction coefficient is demonstrated in Figure 11, Figure 12 and Figure 13.

It is evident (see Figure 11) that the samples made with the Starlit structure exhibited better sound absorption characteristics compared to the samples produced with Octagonal, Rhomboid, and Cartesian structures. It was also found that the noise reduction coefficient generally increased with an increase in the air space size (see Figure 12), the volume ratio (see Figure 13), and the ASB sample thickness (see Figure 11, Figure 12 and Figure 13).

#### 3.2.3. Influence of Volume Ratio

**Figure 14:**
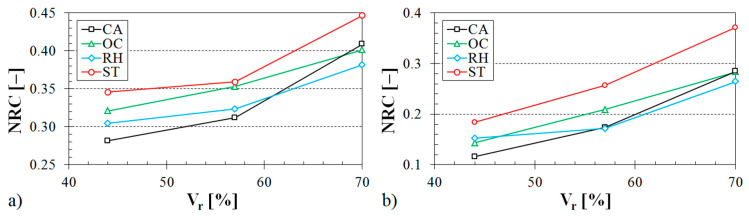
Influence of the 3D-printed ABS material structure on the noise reduction coefficient vs. volume ratio for the investigated ABS samples: (**a**) sample thickness *t* = 3 cm, air space size *a* = 10 cm; (**b**) sample thickness *t* = 1 cm, air space size *a* = 4 cm.

**Figure 15:**
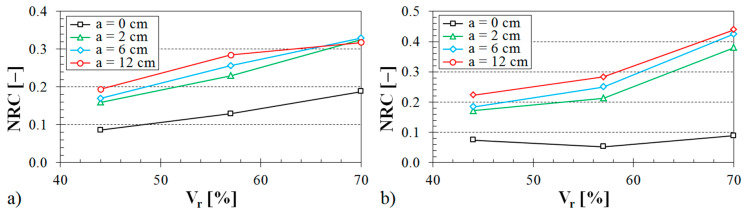
Influence of the air space size on the noise reduction coefficient vs. volume ratio for the investigated ABS samples: (**a**) Rhomboid structure, sample thickness *t* = 2 cm; (**b**) Starlit structure, sample thickness *t* = 1 cm.

**Figure 16:**
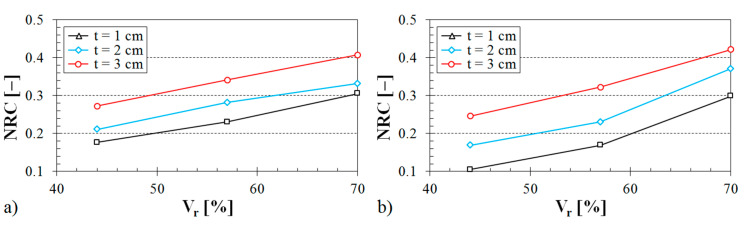
Influence of the sample thickness on the noise reduction coefficient vs. volume ratio for the investigated ABS samples: (**a**) Cartesian structure, air space size *a* = 8 cm; (**b**) Octagonal structure, air space size *a* = 12 cm.

The influence of the ABS volume ratio on the noise reduction coefficient is depicted in Figure 14, Figure 15 and Figure 16.

It was found again that the Starlit-shaped specimens made from the ABS material had a higher capability to dampen noise in comparison with the other structure types (see Figure 14). It is also evident that the volume ratio (see Figure 14, Figure 15 and Figure 16), air space size (see Figure 15), and sample thickness (see Figure 16) had a positive effect on the noise reduction coefficient and, thus, on the sound absorption performance of the tested 3D-printed ABS materials.

## 4. Conclusions

The sound absorption performance of 3D-printed open-porous ABS samples, which were produced with Cartesian, Octagonal, Rhomboid, and Starlit structures, have been evaluated in this work. The investigated ABS samples were also produced with different volume ratios and thicknesses. 

It can be concluded that the Starlit-shaped specimens made from the ABS material exhibited better sound absorption performance in comparison with the other structure types. This was caused by the more complex pore shapes of the Starlit structure, which led to a higher airflow resistivity during the propagation of acoustic waves through this porous structure and, subsequently, to a more efficient conversion from sound energy into heat. It was also found in this work that the sound absorption properties of the tested 3D-printed ABS specimens generally increased with an increase in the sample volume ratio and the material thickness. However, the increasing sample thickness generally results in higher manufacturing costs of the 3D-printed materials. Hence, this method is not effective for sound absorption. Therefore, it is more suitable to use open-porous sound absorbing materials with air spaces in order to improve the sound absorption performance of the ABS materials. Generally, the increasing air space size between the tested sample and the solid wall inside the impedance tube led to a shift of the primary sound absorption maxima to lower excitation frequencies. For this reason, increasing the air space size is suitable for damping of low-frequency acoustic waves. The above findings were also confirmed by the noise reduction coefficient, which expresses an average sound absorption performance of the investigated materials independently of the excitation frequency.

In future work, it is possible to develop and optimize new 3D printing open-porous materials (e.g., more complex pore shapes and advanced multilayer 3D-printed structures) in order to increase acoustic absorption efficiency in a wide frequency range.

## Figures and Tables

**Table 1:** Characteristics of the investigated 3D-printed structures.

Structure Type	Volume Ratio (%)	Label	Front View	Strut Diameter (mm)	Basic Cell Sizes *x*/*y*/*z* (mm)
Starlit	44	ST_44	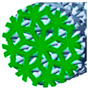	1	9/9/8
	57	ST_57		1.4	9/9/7.5
	70	ST_70		1.8	9/9/8
Rhomboid	44	RH_44	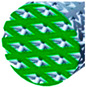	1	7/7/5.5
	57	RH_57		1.35	7/7/5
	70	RH_70		1.7	7/7/5
Cartesian	44	CA_44	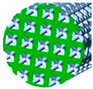	1	5/5/5
	57	CA_57		1.4	5/5/5
	70	CA_70		1.8	5/5/5
Octagonal	44	OC_44	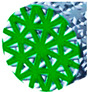	1	7/7/6
	57	OC_57		1.4	7/7/6
	70	OC_70		1.7	7/7/5.5

**Table 2:** Maximum values of the sound absorption coefficient and their corresponding excitation frequency, volume ratio, sample thickness and air space size for the investigated ABS samples with different structure types.

Structure Type	*α_max_* (−)	*f_max_* (Hz)	*V_r_* (%)	*t* (cm)	*a* (cm)
Cartesian	0.999959	3736	70	3	6
Octagonal	0.999995	3488	70	3	8
Rhomboid	0.999763	1728	70	3	10
Starlit	0.999937	3920	70	2	10
